# Supplementary material for: Pentagonal photonic crystal mirrors: scalable lightsails with enhanced acceleration via neural topology optimization
Source: Nat Commun. 2025 Mar 24;16:2753. doi: 10.1038/s41467-025-57749-y (PMC11933360; doi:10.1038/s41467-025-57749-y)
Supplement: Supplementary file 1 — Supplementary Information [file 41467_2025_57749_MOESM1_ESM.pdf]

Supplementary Information on "Pentagonal Photonic Crystal Mirrors: Scalable Lightsails with Enhanced Acceleration via Neural Topology Optimization"

L. Norder, S. Yin, M. H. J. de Jong, F. Stallone, H. Aydogmus, M. A. Bessa, R. A. Norte

February 13, 2025

Section A - Design analysis

24 evaluation points are used in calculating the D in the optimization to reduce the computational cost, resulting in an error to the actual D. Therefore, the three designs from Fig. S1 closest to the 500 nm mean feature size (MFS) objective are studied more thoroughly to choose a final design for this study. Firstly, the full reflectivity spectrum of the four designs is calculated for 300 wavelengths and presented in Fig. S1b. These spectra are then used to integrate the equation of motion directly [4], resulting in the velocity and the travelled distance of the lightsails following the Starshot parameters (Fig. S1d,e), giving insight into how the reflectivity spectrum translates to the D. It stands out that for designs with similar D the actual acceleration time can vary significantly.

Secondly, the design polarisation dependency is studied by plotting the D of the sail for a single plane wave normal to the sail surface by varying from $\phi = 0$ to $\phi = \pi$, because during the launch the lightsail will only be illuminated by a single linear polarised plane wave. Fig. S1c shows that the three designs perform differently. Whereas the D varies negligibly for the hexagonal design, the square design loses all its performance between $\phi = 0.5\pi$ and $\phi = \pi$. Therefore, the feasibility of PhC design depends on the alignment accuracy or the envisioned operating state (e.g. spinning lightsails).

It should be noted that the final acceleration time and distance presented in Fig. S1e are probably not feasible for a physical lightsail, as it is not considering the connection of the sail to the payload. Although this study has shown some practical limits to the lightsail missions, it would be valuable to study the limits of

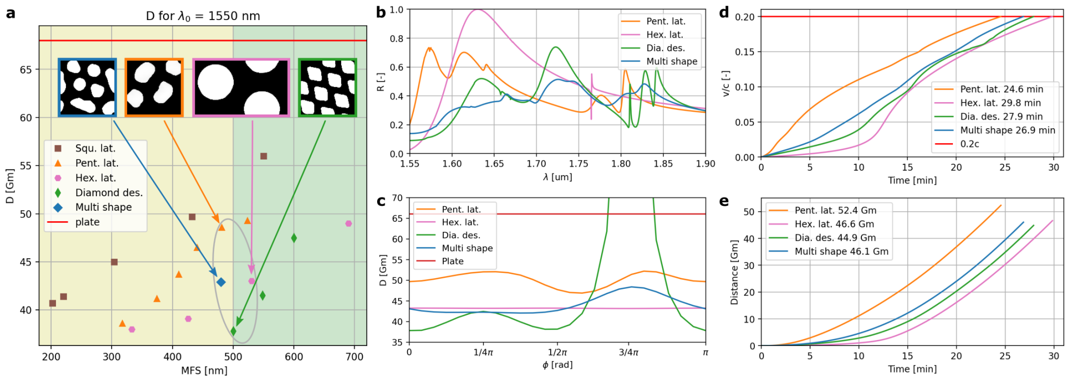

Fig. S 1: Performance evaluation of the obtained designs. **a**, Acceleration distance D of obtained designs for various MFS. **b**, Reflectivity spectrum. **c**, D for one incident plane wave with changing polarisation angle. The velocity (**d**) and the travelled distance (**e**) of a PhC lightsail over its acceleration time.

the different subsystems (e.g. laser array and lightsail) to improve the optimization and feasible design generation. Moreover, Fig. S1c,d shows that optimizing for D alone is insufficient to capture the challenging mission criterion.

To summarise, optimising a lightsail for D needs to consider designs with varying MFS, polarisation dependence, and acceleration time. These three parameters can highly influence the mission's success by determining the fabrication and the launch costs.

Section B - Polarization dependency of final designs

Fig. S2 shows the polarization dependence of the designs from Fig. 4. From this figure, it can be seen that the pentagonal design is more sensitive to the change in polarization compared to the hexagonal. Therefore, the reflectivity spectrum presented in Fig. 6(c) will result in a slightly different spectrum when measured with an orthogonal polarization.

Section C - System costs optical lithography

Multiple optical lithography systems with different light sources and, therefore, different minimum line widths are presented in Supplementary table 1. However, the MFS of this lithography method will exceed the minimum line width (MLW) due to the diffraction limit of the light. Therefore, sharp features like curvatures may not be captured during production, resulting in an MFS of 500 nm. For this study, i-line photolithography was selected based on its cost-effectiveness, availability, and established processing protocols.

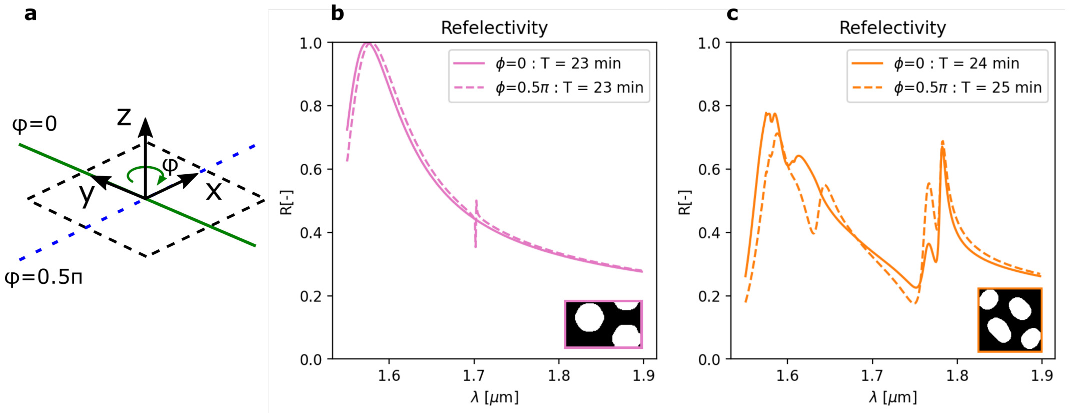

Fig. S 2: Polarization dependency of the final optimized pentagonal and hexagonal PhC design. **a**, polarization orientation of incident plane wave. The polarization dependency of the reflectivity spectrum for hexagonal (**b**) and pentagonal (**c**) design.

Source	Spectrum	λ[nm]	MLW [nm]	system cost [$M]
Hg i-line	UV	365	350	4-6
KrF	DUV	248	150	7-11
ArF	EUV	193	19	25-110

Supplementary table 1: Minimal line width (MLW) for optical lithography using different light sources [5, 1]

Section D - Measurement simulations

To accurately represent the measurement, a new simulation is performed, in which not only the membrane in a vacuum but also the Si wafer and the gap between the wafer and the membrane are simulated. Additionally, a small layer with relative permittivity between air and Si is added to simulate the roughness of the Si substrate due to the fabrication. Fig. S3 shows a representative measurement that is fitted to the model by varying the height of the SiN layer, the gap between the Si and SiN, and the layer representing the rough Si surface.

When measuring from the inside of the membrane to the outside, a change in the measured spectrum is observed. Fig. S4 shows four measurements from the center to the edge of the membrane. By fitting the measurement to our model, we think that the main contribution to the change in the measurement is the thickness change over the membrane, which comes from the position-dependent etch rate.

Section E - Mass constraint

In the context of the Starshot initiative, the total mass of the lightsail has been suggested to be 1 gram. The design we found, as reported in the main text and illustrated in Fig. 3, has an approximate mass of 3 grams for a 10 m^2 lightsail using

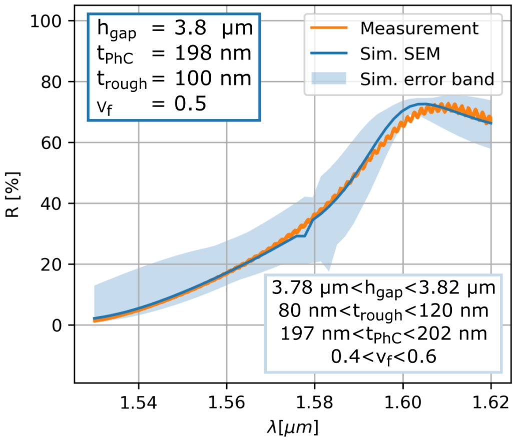

Fig. S 3: Reflectivity measurement from the 60×60 mm^2 suspended photonic crystal membrane together with the RCWA simulation of the reflectivity and related error band. h_{gap}, the height of vacuum gap between the membrane and the Si substrate; t_{PhC}, the thickness of the membrane; t_{rough}, the thickness of the roughness layer in the simulation; v_f, the volume fraction of the roughness layer which determines the relative permittivity of the layer by $\epsilon_{rough} = \epsilon_{Si} v_f + \epsilon_{SiN}(1 - v_f)$ [2].

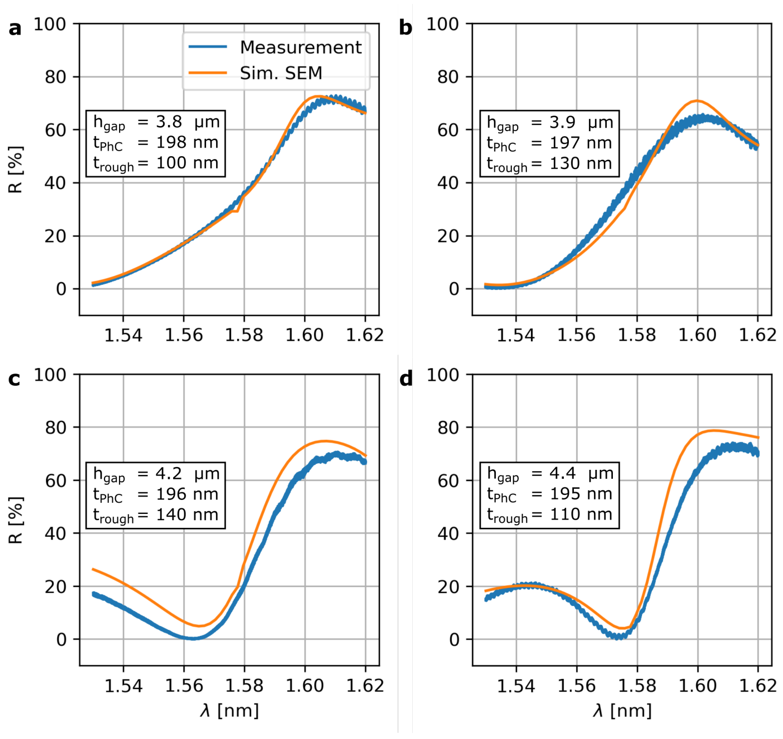

Fig. S 4: Reflectivity spectrum measured (blue) from the center to the edge of the sample together with the simulation (orange) of the 60×60 mm^2 suspended PhC membrane. For each measurement, the fitting parameters are presented

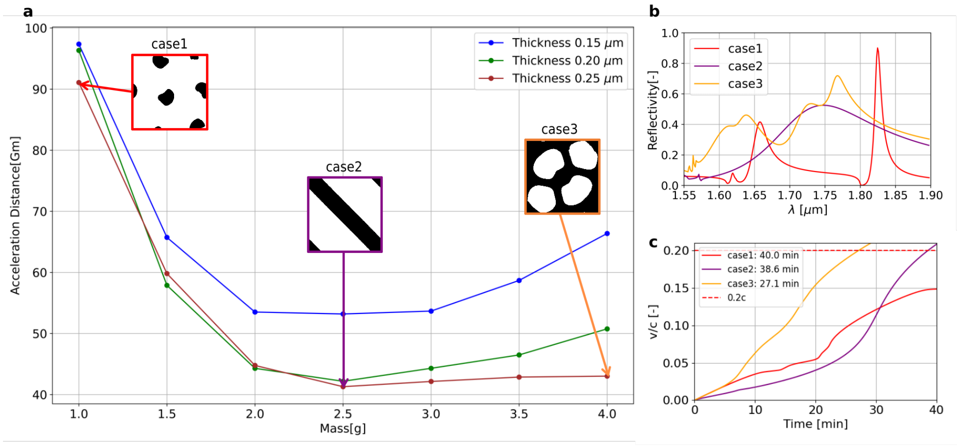

Fig. S 5: Best design for different mass constraints. **a**, Limiting the lightsail to 1 gram precludes manufacturability, as shown in Case 1. Case 2 yields the shortest acceleration distance yet suffers from significantly reduced reflectivity compared to Case 3, which exhibits a marginally greater acceleration distance. **b**, Reflectivity spectrum for these three cases. Case 3 has a relatively lower acceleration distance but the highest reflectivity. **c**, Case 3 has the lowest acceleration time because of the high reflectivity.

the selected material. We have investigated the feasibility of creating a lightsail with less mass by adjusting the area fraction and the sail's thickness, ensuring a particular total mass value is satisfied. Fig. S5 illustrates the outcome of the optimization for different mass constraints when considering different sail thicknesses. We found that the designs obtained for a mass constraint of 1 gram are not viable for manufacturing (labelled case 1 in the figure) because they have freely suspended masses. In fact, the main factor contributing to a large acceleration distance is the low mass of these sails, rather than an increase in reflectivity. We also highlight that the best designs we found for a lightsail with 2.5 grams are still not viable for manufacturing, as they would correspond to parallel strings of material – case 2 in the figure. Furthermore, we show in part Fig. S5b,c that the lower reflectivity across the laser wavelength spectrum leads to a higher acceleration time, which would require more exposure to the laser light. Therefore, the pentagonal crystal structure found for larger mass values is more interesting, as it has lower acceleration time and it is viable for manufacturing.

Section F - Neural topology optimization

In this study, we investigated for the first time the use of neural topology optimization in Photonics by considering a convolutional neural network that reparameterizes the lightsail design on the fly (without training). This recent method has been evaluated

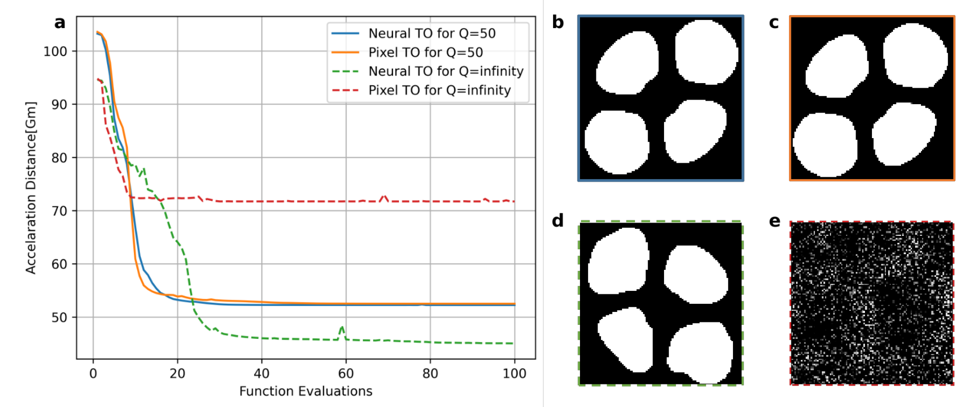

Fig. S 6: Comparative performance of the "Pixel" and neural TO models on a fabricated unit cell with a periodicity of 3.0 μm and thickness of 200 nm. **a**, Loss curves for the two models, with and without the relaxation factor. **b** and **c** show the similarity in design and objective values achieved by the "Pixel" TO model and neural TO model, respectively, when a relaxation factor is applied. Without the relaxation factor, as shown in panels **d** and **e**, the "Pixel" model does not converge to a good solution, whereas the neural TO model successfully converges.

.

for structural optimization tasks, but we believed that it would be more advantageous in the context of lightsail design because conventional topology optimization requires a relaxation factor Q to ensure a smooth optimization process [3]. The conventional strategy utilizes a pixel-based model and the Method of Moving Asymptotes (MMA) as the optimizer. We implemented this strategy using the NLopt library, referring to it as "Pixel model", and compared it with the neural TO method.

In the absence of a relaxation factor the simulation is closer to the real physical problem, but the conventional TO method does not converge to a good solution. Even when considering more than 100 iterations, there is virtually no change in the design for the conventional strategy. In contrast, our findings demonstrate that the neural TO method finds a similar solution (pentagonal crystal) without the need for this relaxation factor, while predicting a lower (better) objective value. The reparameterization technique allows to handle objective landscapes that are non trivial and non-convex by over-parameterizing the problem and avoids getting stuck in local optima. We found this method to be robust and we did not have to invest significant time in hyperparameter optimization. We believe that neural TO will open new avenues in design for problems that may involve even more complex objective landscapes in the future.

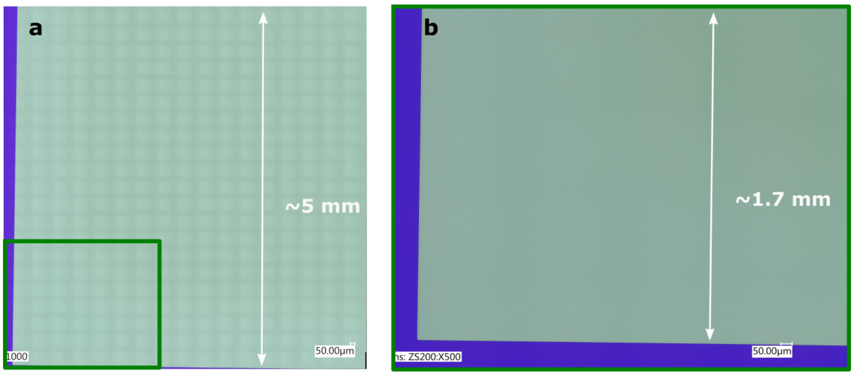

Fig. S 7: **a**, Stitched microscopy image of the corner of the 60×60 mm^2 PhC membrane. Shows a microscopy image where multiple images with $1000\times$ magnification are stitched together. The hatch pattern on the image is due to the uneven lighting of the microscope. In this figure, we do not see the interference pattern as we have an objective with a small depth of field, confirming that the membrane is not wrinkled. **b**, one image of the corner of the membrane with $500\times$ magnification.

Section G - Surface of the PhC membrane

A study was performed to investigate the origins of the light interference pattern seen in Fig. 5a. We think it does not come from membrane wrinkling but from reflections on the uneven substrate surface beneath the semi-transparent membrane. This pattern is due to the substrate's inherent roughness and the slightly varying gap created by the membrane's undercut.

First, a microscopy image was taken of the corner of the membrane, presented in Fig. S7. The observed color in a microscopy image is highly sensitive to any nanometer-scale wrinkles, which would appear as dark features if present. Unlike standard photography, microscopy allows nanoscale precision in focusing directly on the membrane, giving a true sense of color and flatness. The uniform color and absence of dark wrinkles in these images indicate that the membrane is wrinkle-free.

Secondly, the white light interferometric measurements shown in Fig. 2a-d are performed to provide a more detailed look. At low magnification ($2\times$), interference from substrate reflections is visible; however, at $20\times$ magnification (Fig. 2d), these reflections disappear, confirming that the interference pattern originates from the substrate rather than wrinkles in the membrane. This difference in measurements across magnifications further supports that the membrane itself is smooth and wrinkle-free.

Additionally, our deposition process induces a tensile stress of 270 MPa, which greatly contributes to membrane flatness. This stress is comparable to stress induced in duct tape if a grand piano were hung off of it; this level of tension ensures that the remaining membrane is extremely flat.

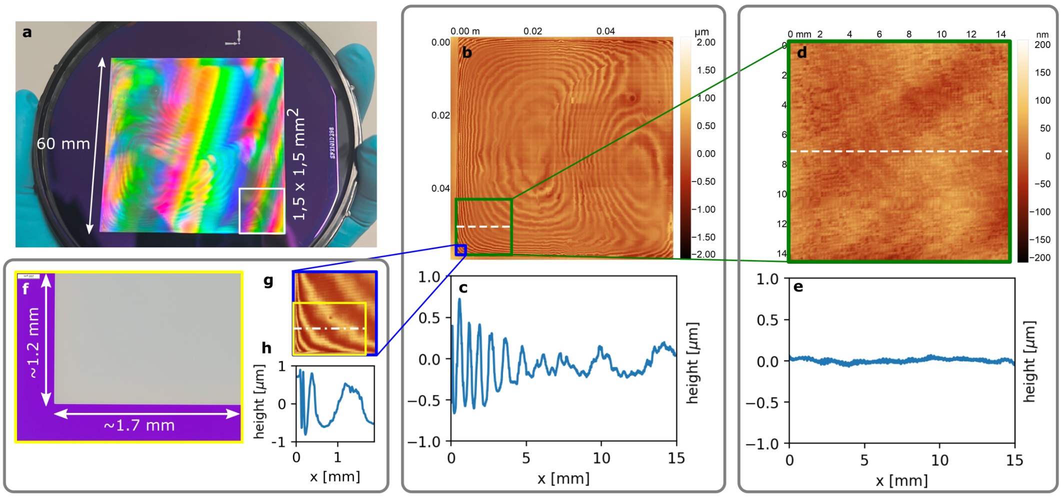

Fig. S 8: **a**, Photograph of the 60×60 mm^2 PhC membrane. **b**, height map of full wafer measured with Bruker white light interferometer measured at $2\times$ magnification. **c**, height measurement over the white line shown in (**b**). **d**, height map of the green region, measured at $20\times$ magnification. **e**, height measurement over the white line performed at $20\times$ magnification. **f**, microscopy image of the corner of the membrane (yellow). **g**, zoom in on the corner of the measurement shown in (**b**)(blue). **h**, height measurement over the white line in the blue region (**g**).

References

[1] Robert Castellano. The switch to asml's euv lithography will impact the entire semiconductor supply chain. https://seekingalpha.com/article/4059013-switch-to-asmls-euv-lithography-will-impact-entire-semiconductor-supply-cha Accessed: 2023-03-03.

[2] Hiroyuki Fujiwara. *Effect of Roughness on Ellipsometry Analysis*, pages 155–172. Springer International Publishing, Cham, 2018.

[3] Weiliang Jin, Wei Li, Meir Orenstein, and Shanhui Fan. Inverse design of lightweight broadband reflector for relativistic lightsail propulsion. *ACS Photonics*, 7(9):2350–2355, September 2020.

[4] Neeraj Kulkarni, Philip Lubin, and Qicheng Zhang. Relativistic spacecraft propelled by directed energy. *The Astronomical Journal*, 155(4):155, mar 2018.

[5] Ekta Sharma, Reena Rathi, Jaya Misharwal, Bhavya Sinhmar, Suman Kumari, Jasvir Dalal, and Anand Kumar. Evolution in lithography techniques: Microlithography to nanolithography. *Nanomaterials*, 12(16), 2022.
